# Using energy psychology to remediate emotional wounds rooted in childhood trauma: preliminary clinical guidelines

**DOI:** 10.3389/fpsyg.2023.1277555

**Published:** 2023-10-18

**Authors:** David Feinstein

**Affiliations:** Independent Practitioner, Ashland, OR, United States

**Keywords:** adverse childhood experiences, developmental trauma, emotional freedom techniques, energy psychology, somatic therapy, thought field therapy

## Abstract

Adverse childhood experiences (ACEs) are potentially traumatic events that occur in childhood, such as violence, abuse, severe neglect, or mental health problems in caregivers. The negative physical and mental health consequences of severe or multiple ACEs provide a major challenge for the health care community. Psychotherapies that utilize a mind–body approach in treating ACE-related conditions are seen by their proponents as having advantages for bringing healing and restoration compared with talk, introspective, interpersonal, and exposure therapies that do not intervene at the body level, as famously encapsulated by Bessel van der Kolk’s observation that “the body keeps the score.” A mind–body approach whose use has been rapidly increasing in clinical settings as well as on a self-help basis is called “energy psychology.” Energy psychology combines conventional therapeutic techniques such as cognitive restructuring and psychological exposure with the stimulation of acupuncture points (acupoints) by tapping on them. A review of the development, efficacy, and plausible mechanisms of energy psychology is presented, and several strengths are enumerated, such as how integrating acupoint tapping into conventional exposure methods enhances the speed and power of outcomes. The impact of energy psychology protocols on the three brain networks most centrally involved with ACEs is also examined. Finally, recommendations are offered for using an energy psychology approach at each stage of therapy with individuals who have endured severe or multiple ACES, from establishing a therapeutic alliance to assessment to treatment to follow-up.

## Introduction

A questionnaire responded to by 9,508 Kaiser Permanente patients found correlations between adverse childhood experiences (ACEs) and subsequent physical health, mental health, and lifestyle difficulties (Felitti et al., 1998), correlations that have been corroborated in subsequent studies (Hughes et al., 2017). Categories of ACEs identified in a frequently used self-report include emotional, physical, and sexual abuse; emotional and physical neglect; and household dysfunction, which might involve parental separation/divorce, violence against the mother, household substance abuse, household mental illness, or incarceration of household member (Cheong et al., 2017). Subsequent categories of ACEs associated with harmful consequences include experiences of social disadvantage, economic hardship, sudden loss of a parent, bullying, discrimination, medical trauma, community violence, and natural disasters (Bartlett and Sacks, 2019). The detrimental physical and mental health consequences of severe or multiple ACEs are well-established and constitute an ongoing challenge for health care professionals (Asmundson and Afifi, 2019). Survey data project that approximately 61% of adults in the United States have experienced one category of ACE and 17% have experienced four or more (Centers for Disease Control and Prevention, 2023).

Undergoing multiple ACEs has a cumulative negative effect on health outcomes and life quality (Lanier et al., 2018). The original study found that “the number of categories of adverse childhood exposures showed a graded relationship to the presence of adult diseases including ischemic heart disease, cancer, chronic lung disease, skeletal fractures, and liver disease” (Felitti et al., 1998, p. 245). Exposure to four or more categories “had 4–12-fold increased health risks for alcoholism, drug abuse, depression, and suicide attempt” (p. 245). Among other long-term mental health consequences of severe or multiple ACEs are increased incidence of anxiety, PTSD, sleep disturbances, and learning difficulties (Asmundson and Afifi, 2019). Additional medical conditions that are more frequently seen include diabetes, obesity, HIV, and changes in brain development that affect the way the body responds to stress. Also identified were greater occurrences of injuries, unsafe sex, sexually transmitted infections, maternal and child health problems, homelessness, criminality, and involvement in sex trafficking.

The consequences of ACEs can become biologically embedded during critical stages of development, potentially resulting in enduring physical and mental health problems throughout life (Berens et al., 2017). Because of the wide variety of ACEs, and because severe or multiple occurrences lead to such a broad range of adult diseases and mental health risks, any treatment strategy must be particularly flexible in adapting itself to the individual’s unique history and temperament. History is obviously foundational. For instance, while undergoing multiple ACEs has a clear association with greater incidences of psychological and physical health disorders, a single traumatic experience such as witnessing the murder of a parent may have lifelong consequences. The critical role of temperament is illustrated by Bartlett and Sacks (2019) observation that “two children who experience the same type of adversity may respond in distinct ways: One may recover quickly without significant distress, whereas another may develop posttraumatic stress disorder” (para 5).

Outcome evidence following therapeutic interventions with individuals who have experienced severe or multiple ACEs is equivocal (Lorenc et al., 2020). Psychodynamic therapy, family therapy, and motivational interviewing have been found to be relatively ineffective, with the most promising results coming from CBT. ACEs often lead to “complex needs, but there is limited information in the evaluation literature about how best to address these needs” (Lorenc et al., 2020, para. 15).

In an empirically informed workbook for individuals who self-identify as having been impaired by their ACEs, Schiraldi (2021) is encouraging:

Can negative neural patterns be rewired? The short answer is yes, because the brain is plastic, capable of changing and rewiring neural circuitry. However this does not usually happen through insights or understanding alone. Memories of toxic stress play out not so much in the thinking and verbal regions of the brain, but in the visual emotional and visceral parts of the brain…Rewiring these memories requires strategies that are deeper than insights and understanding…What heals the traumatized brain is the felt sense of love, safety, and calm which register…in areas of the brain that words and thinking do not touch (p. 78).

Consistent with this appraisal, Ranjbar and Erb (2019) recommend a “biopsychosocial” model for “trauma-informed care” that includes

mind–body skills practices such as breath and body awareness, neuromuscular relaxation training, cognitive behavioral integration, mindfulness and meditation training, biofeedback, guided imagery, Qi Gong, or yoga [which] provide physiological mechanisms of regulation and resilience without a need to directly address specific psychological content (para. 22).

*Energy psychology* is a mind–body approach that combines physiological mechanisms of regulation with a capacity for directly addressing specific psychological content. This paper will briefly review the history of energy psychology, its efficacy data, postulated mechanisms, strengths for altering neural networks that are functionally impaired by severe or multiple ACEs, and finally turn to proposed clinical guidelines.

## The emergence of energy psychology

The most widely practiced and systematically researched form of energy psychology is “the Emotional Freedom Techniques” or simply EFT (Church, 2018). EFT is a derivative of Thought Field Therapy or TFT (Callahan, 2002). Both approaches build upon established clinical methods, such as psychological exposure and cognitive restructuring while adding the stimulation of selected acupuncture points (acupoints) by tapping on them. Mounting evidence suggests that acupoint tapping sends clinically beneficial activating and deactivating signals to brain areas that were aroused during the cognitive and exposure aspects of the protocol (Feinstein, 2023). The core hypothesis for explaining energy psychology’s consistently strong clinical outcomes is that its procedures generate electromagnetic signals that have a therapeutic effect on areas of the brain involved with distress, trauma, or mental illness (Feinstein, 2023).

Energy psychology was initially met by the clinical community with strong skepticism and sometimes ridicule about the odd-looking procedures and initial lack of empirical evidence or even a plausible explanatory framework (e.g., Gaudiano and Herbert, 2000; McNally, 2001). Yet its use and acceptance have been escalating. A credible estimate by Leskowitz (2016) has placed the number of therapists using energy psychology in the “tens of thousands” (p. 181). Meanwhile, a comprehensive research review of EFT by Church et al. (2022) also emphasizes the self-help as well as clinical applications of the approach. Church et al. estimate that “tens of millions” of people use energy psychology worldwide, noting that one EFT phone app alone has had more than 2 million documented users.

## Efficacy

A systematic assessment of peer-reviewed papers on the topic of energy psychology (Feinstein, 2023) identified 309 entries published in English language journals through February 2022. Of these, 125 were clinical trials investigating protocols that included acupoint tapping (i.e., TFT or EFT or their variations). Of those, 69 were randomized controlled trials (RCTs), with the other 56 being outcome studies not utilizing a comparison condition. Of the 125 clinical trials, 123 showed statistically significant improvement on standardized pre/post-measures. This assessment corroborated a comprehensive review of more than 100 randomized controlled trials and outcome studies of EFT (again, the most popular form of energy psychology) which concluded that “comparatively few treatment sessions are required, treatment is effective whether delivered in person or virtually, and symptom improvements persist over time” (Church et al., 2022, p. 2).

### Meta-analyses

Meta-analyses focusing on the use of acupoint tapping protocols have found strong effect sizes (above 0.8) on some of the most common and serious mental health sequelae of severe or multiple ACEs, including depression, anxiety, and PTSD. Clond (2016) reviewed 14 RCTs reporting the use of EFT in treating anxiety (total of 658 participants). The overall pretreatment to posttreatment effect size was 1.23. In the treatment of depression using EFT, Nelms and Castel (2016) found a pretreatment to posttreatment effect size of 1.85 based on 12 RCTs with a total of 398 participants. Investigating six studies of EFT in the treatment of PTSD (295 participants), Stapleton et al. (2023) found the pretreatment to posttreatment effect size to be 1.86. While the depression and anxiety meta-analyses are out of date and include some RCTs whose designs are of low quality, they are the most recent meta-analyses of the two conditions and their conclusions are consistent with the findings of more recent and well-designed RCTs.

### Rapid improvement

While clinical trials applying energy psychology specifically to ACEs have not been conducted, of the many types of ACEs that might impact a child’s development, few can be more damaging than living as an orphan after having watched one’s parents or neighbors being murdered. A dozen years after the 1994 genocide in Rwanda, an acupoint tapping approach was brought to 50 orphans at the El Shaddai Orphanage in Kigali, Rwanda (Sakai et al., 2010). These adolescents were selected after evaluations showed that they still suffered with severe symptoms of PTSD, including anxiety, depression, flashbacks, nightmares, and insomnia. Many had witnessed their parents or other relatives or neighbors being slaughtered. The outcomes of the treatment were measured using an established PTSD symptom checklist completed by the orphans’ caregivers before and following the single-session treatments. The test was translated from English into the local language, Kinyarwanda, in collaboration with its developers.

Improvements vastly exceeded those of *any* previous peer-reviewed study of a PTSD treatment in terms of speed, degree of effectiveness, and percentage of subjects who were helped. After a single tapping session using Thought Field Therapy, 47 of the 50 orphans were no longer in the PTSD range on the standardized assessment. Benefits were sustained on a 1-year follow-up using the same symptom checklist, again based on the caregiver’s observations. These paralleled findings from pre/post-treatment symptom checklists completed by the adolescents.

While the single-session remediation of symptoms reported in the above study is unusual and may seem improbable, and further study is certainly required before any firm conclusions can be drawn, three other clinical trials have also shown pronounced reduction of PTSD symptoms after a single tapping session (Church et al., 2011; Connolly and Sakai, 2011; Connolly et al., 2013). The meta-analysis of six studies using EFT in the treatment of PTSD of Stapleton et al. (2023) found a large effect size (Hedge’s *g* = 1.86) after between 4 and 10 sessions. For comparison, the average number of CBT treatments recommended for addressing PTSD is generally between 8 and 15 sessions (Watkins et al., 2018).

### Comparisons with other therapies

Systematic reviews or meta-analyses that have compared the outcomes of different psychotherapies administered after disasters or other forms of severe mental distress have revealed a wide range in the effectiveness among the different methods examined. Both reductions in trauma-related symptoms and enhancements in overall functioning are generally tracked. The majority of approaches investigated were variations of Cognitive Behavioral Therapy (CBT), such as Narrative Exposure Therapy (NET), and a trauma-focused form of CBT (TF-CBT). CBT is often regarded as the “Gold Standard” for treating severe psychological conditions (David et al., 2018). However, other therapies were also included in the comparisons, such as meditation, play therapy with children, family therapy, and Eye Movement Desensitization and Reprocessing (EMDR).

Six of these studies (Brown et al., 2017; Morina et al., 2017; Purgato et al., 2018; Bangpan et al., 2019; Mavranezouli et al., 2020; van Ginneken et al., 2021) included TFT or EFT in their comparisons. Both approaches demonstrated significant positive outcomes and favorable comparisons with other therapies. For example, Brown et al. (2017) conducted a meta-analysis of psychological treatments for children who had experienced trauma resulting from either manmade or natural disasters. Among the 36 studies analyzed, only one utilized an energy psychology approach, TFT. The effect sizes ranged from 0.09 (indicating a small effect) to 4.19 (indicating an extremely large effect). The average effect size from pre-treatment to post-treatment across all groups was 1.47, which is considered a large effect. The largest effect size observed among the investigated treatments, 4.19, was achieved by TFT. Brown et al. note, however, that TFT utilizes elements found in other therapies that were included in the comparisons and that the evidence for it is limited because only a single TFT study met the inclusion criteria.

For this reason, the single tapping study considered in Brown et al. might have been an outlier. Another comprehensive meta-analysis, in which 32 studies investigated 17 different interventions for treating traumatized youth with PTSD, lends corroboration (Mavranezouli et al., 2020). In this comparison, EFT was one of the two statistically most effective of the 17 therapies in reducing PTSD symptoms at the end of treatment, and it was statistically the most effective intervention in maintaining improvements in PTSD symptoms on follow-up. The authors emphasize, however, that this large positive effect is based on very limited evidence and that trauma-focused CBT has the strongest overall efficacy evidence of the treatments reviewed.

Ten head-to-head studies have compared tapping therapies directly with CBT. Eight of these were RCTs. All 10 found at least equivalent outcomes and, in several of the comparisons, the energy psychology protocols outperformed CBT in speed and durability on follow-up (reviewed in Feinstein, 2022). Comparing CBT and energy psychology, Mollon (2008) concluded that energy psychology is not an alternative to CBT, but a “crucial additional component that greatly enhances its efficacy,” providing more effective means for “affect regulation, desensitization, and pattern disruption” (p. 341). While a weakness of the head-to-head studies is that the investigators in many of them were advocates of energy psychology, in neither the Brown et al. nor the Mavranezouli et al. comparative meta-analyses were any of the investigators proponents of the approach, nor did they give particular attention to the tapping therapies beyond listing their high effect sizes.

## Conditions treated

Psychological conditions identified in the Feinstein (2023) review as having responded to acupoint tapping treatments include anxiety, depression, PTSD, phobias, anger, concentration difficulties, food cravings, insomnia, and performance blocks. A range of physical conditions also showed improvement, including fibromyalgia, chronic pain, headaches, frozen shoulder, psoriasis, obesity, immune function, inflammation, and cardiovascular function. Statistically significant improvements with this broad range of physical diagnoses may be of particular interest when considering the health consequences of severe or multiple ACEs. For conditions such as psychotic disorders, dementia, autism, bipolar, or deeply ingrained personality disorders, although improvements in coping and comfort have been reported anecdotally, no clinical trials investigating reversals of these conditions were identified in the review.

## Durability

Of the 125 clinical trials reviewed in Feinstein (2023), 81 conducted follow-up investigations (including 50 of the RCTs and 31 of the uncontrolled trials). Of these, 79 of the 81 found that the benefits were sustained. “Benefits sustained” was defined as a statistically significant (*p* < 0.05) improvement between the pre-treatment assessment and the assessments at the end of the follow-up period on at least one of the major targets for change. The most frequent follow-up periods ranged from 1 month to 2 years, with a mean of 7 months.

## Mechanisms

An obstacle to the acceptance of energy psychology by the clinical community has been that claims of potent and rapid clinical improvement were presented long before scientifically plausible explanations appeared for how the admittedly odd-looking procedures might work. For instance, since tapping protocols utilize cognitive restructuring and psychological exposure plus other features common to all therapies such as client expectation and therapeutic alliance (Feinstein, 2023), criticisms of the method (e.g., Gaudiano et al., 2012; Bakker, 2013) have suggested that acupoint tapping is an inert tangential procedure rather than an essential ingredient for producing the strong outcomes reported in the efficacy literature.

Seven studies have investigated this question (Feinstein, 2023). In the most recent and stringent of these, 88 women suffering with mild to moderate depression were randomly assigned to an EFT group or a control group that used identical treatment procedures except that the acupoints were replaced by sham points (Mehdipour et al., 2021). While both groups showed significant decreases in depression on the Beck Depression Inventory after 8 weeks of otherwise identical treatment, the improvements in the group that used the recognized acupuncture points were far stronger (*p* < 0.001) than in the group that used the sham points. Five of the six earlier studies investigating whether acupoint tapping is an essential ingredient also concluded that the acupoint tapping is an active and necessary ingredient for bringing about the demonstrated clinical outcomes.

While mechanisms that explain the actions of acupoint tapping protocols are still being investigated, several empirically supported premises have been formulated that account for many of the clinical improvements described in the literature (Feinstein, 2021b). Among them:

1. Tapping on an acupuncture point generates an electrical impulse.
2. These impulses function as activating and deactivating signals when they reach brain regions involved with the condition being treated.
3. The words and images used *during* the tapping activate brain regions that attract the signals generated *by* the tapping to those regions.
4. Neural changes make the benefits durable.
5. Other biological shifts explain health benefits beyond targeted issues.

More details on each follow.

### Tapping on an acupuncture point generates an electrical impulse

Certain large proteins within the membranes of skin cells convert mechanical pressure into electrical impulses in a well-established process called mechanosensory transduction (Bagriantsev et al., 2014). Mechanosensory transduction produces *piezoelectricity* (literally, “electricity caused by pressure”) and can be initiated by tapping, massaging, or inserting a needle into an acupoint. The skin conducts electricity due to the presence of electrolytes such as sodium, potassium, and chloride ions in sweat and other bodily fluids, but acupoints are more efficient producers of piezoelectricity than adjacent areas of the skin because of their distinguishing electrochemical properties, such as less electrical resistance and greater conductivity (Ahn et al., 2008).

The pathways traveled by the electrical impulses generated by stimulating acupoints are situated along the body’s connective tissue (Langevin and Yandow, 2002). Most acupoints are located above aggregates of connective tissue. Connective tissue is composed largely of collagen. Because collagen conducts electricity, afferent signals traveling through the body’s connective tissue can reach the brain more rapidly and reliably than those traveling from neuron to synapse to neuron through the nervous system (Oschman, 2003).

### These impulses function as activating and deactivating signals when they reach brain regions involved with the condition being treated

Stimulating pertinent acupuncture points not only generates electrical impulses that can be rapidly transmitted to distant areas of the body, these impulses are able to stimulate executive regions of the brain or abruptly turn off hyperarousal in the limbic system. A 10-year research program conducted at Harvard Medical School used sophisticated imaging equipment to study the effects of stimulating selected acupuncture points in various brain regions (Fang et al., 2009). The investigators found that certain points can send signals to the amygdala which reduce threat activation almost instantly.

Several imaging studies have been conducted to date that focus specifically on *tapping* acupuncture points rather than using acupuncture needles or electrical stimulation, as were utilized in the Harvard studies. Initial findings suggest that the procedure can generate both activating and deactivating signals that lead to clinically beneficial shifts in brain activity. For instance, in a study of the effective treatment of a fear of flying, magnetoencephalography (MEG) revealed that, as would be expected based on the clinical outcome, the brain’s response to perceived threat when thinking about flying was greatly reduced as subjective symptoms subsided (Di Rienzo et al., 2020). The investigators also found that frontal executive regions—which are responsible for cognitive evaluation and regulating emotional responses to stressful stimuli—were activated.

Corroboration of the findings from this single case was found in a study that used electroencephalography (EEG) to investigate neural changes following acupoint tapping or a progressive muscle relaxation control with 22 subjects who met the criteria for anxiety disorder. Acupoint tapping was more effective in reducing anxiety, downregulating the activity of limbic and cerebellar brain regions implicated in the fear response while increasing the engagement of frontal executive regions that mediate limbic responses to stressful stimuli (König et al., 2019). These studies suggest that acupoint tapping generates signals which increase activity in brain regions involved with emotional self-management while generating deactivating signals that downregulate activity in emotional centers that are in hyperarousal. It is interesting to note that these implications are consistent with the belief in traditional Chinese medicine that acupuncture treatments inherently seek to create a state of balance and homeostasis (Xu et al., 2018).

### The words and images used during the tapping activate brain regions that attract the signals generated by the tapping to those regions

When an area of the brain is active, blood flow to that area increases, as routinely demonstrated in functional magnetic resonance imaging (fMRI) and positron emission tomography (PET) scans. This allows researchers to view what is occurring in the brain when a person is focusing on a particular type of thought—whether love, fear, anger, or jealousy—or when a mood spontaneously shifts. In a tapping session, the client’s words and images generate neural activity in regions of the brain associated with the targeted therapeutic issues (Stapleton et al., 2019, 2022). For reasons not entirely understood, however, these activated brain regions, with their increased blood flow, become like magnets, attracting the electrical impulses produced by the tapping. The signals seem to balance overstimulated emotional centers and increase activation in executive brain regions involved with problem-solving and emotional management (Feinstein, 2023).

By directing a client’s attention during tapping, a therapist may be efficiently, even if unwittingly, targeting the brain regions that will receive the activating or deactivating signals produced by the tapping. The therapist does not need to know that it is the lateral nucleus of the amygdala that is receiving the deactivating signals, or the dorsolateral prefrontal cortex that is being activated, only that the client’s symptoms or goals are brought into conscious awareness. By guiding the client to focus on specific words and images, precise neurological changes can be activated by the tapping.

### Neural changes make the benefits durable

Even if tapping on a set of acupuncture points sends signals that reduce distress about an issue that has been brought to mind, what causes the changes to persist? If a client has a fear of dogs and brings thoughts of a dog to mind while tapping, the tapping presumably reduces the fear response in the emotional centers of the brain and the person will feel relief *during* the tapping. But why would they not experience fear the next time they think about or see a dog? It is one thing to calm an emotion in the moment. Deep breathing can often accomplish this. It is another to reprogram the response to a trigger that provokes fear, sadness, anger, or guilt. Yet follow-up investigations of energy psychology treatments have consistently shown the improvements to be durable, even after brief treatments. As mentioned earlier, 79 of the 81 clinical trials that conducted follow-up investigation found that at least one targeted change was sustained (Feinstein, 2023).

#### Neurological changes that persist following tapping sessions

Brain imaging studies validate not only that the brain areas involved with the issues focused upon during tapping are altered, but also that the changes are lasting. For instance, Stapleton and colleagues conducted separate fMRI investigations of tapping protocols in the treatment of food cravings and of chronic pain. In the food craving study, 15 overweight or obese individuals were shown images of high caloric “junk” food while in an fMRI scanner (Stapleton et al., 2019). Although complex responses to food are not controlled by any single part of the brain, the fMRI scans revealed activation in the *lateral orbito-frontal cortex* (associated with reward) and the superior temporal gyrus (related to perceiving and interpreting nonverbal cues and integrating visual input). The same images of junk foods following eight group sessions utilizing a tapping protocol showed significantly decreased or even no activity in these areas. These fMRI changes corresponded with decreased subjective attraction to the same foods as shown in self-report questionnaires. Many of the participants were not even able to recall that they had craved foods which they had recently been eating every day.

In the pain study, 24 adults suffering with chronic pain participated in a 6-week online group program using a tapping protocol for treating pain (Stapleton et al., 2022). Pre/post-treatment resting state fMRI changes were found in the medial prefrontal cortex (a pain modulating area) and the bilateral gray matter areas in the posterior cingulate cortex and thalamus (both related to the modulation and catastrophizing of pain). These changes correlated with significant decreases in reported pain severity and the degree to which pain interfered with daily activity. In both studies, the neurological changes persisted following the end of treatment.

#### Brain mechanisms that support lasting cognitive changes

Learning that is built upon experiences involving physical threat or emotional harm may become deeply ingrained in the brain, with robust resistance to change. Ecker et al. (2012) explain that “learning accompanied by strong emotion form neural circuits in subcortical implicit memory that are exceptionally durable, normally lasting a lifetime” (p. 8). While these inflexible cognitive structures may initially reflect a child’s resourceful attempts to navigate challenging circumstances, they can later become self-defeating, contributing to the range of mental and physical disorders seen in individuals who have undergone severe or multiple ACEs. Evolution did, however, devise a process by which even deeply embedded emotional learnings can be updated (Lane et al., 2015). The neural coding of a cognitive schema can be “unlocked,” enabling it to be rewritten in accordance with the new learning. In the case of irrational fear, new information that challenges erroneous formulations in the existing fear structure must be accommodated by that structure if the irrational fear is to be altered (Watkins et al., 2018). Energy psychology builds upon this process.

#### How acupoint tapping utilizes these brain mechanisms

If a person brings to mind a food that induces a craving and then stimulates acupoints that reduce the craving, a new experience is created. Thoughts of the food no longer trigger the craving. What is expected does not occur. Such prediction errors—viscerally registered mismatches between expectation and experience—are central to the way cognitive schemas update themselves (Exton-McGuinness et al., 2015). Prediction errors are, in fact, a primary reason a prevailing cognitive schema becomes labile for change. These mismatches are inherent in the process of cognitively accommodating rather than distorting contradictory information. For a prediction error to dislodge an existing cognitive structure, however, it must “feel decisively *real* to the person based on his or her own living experience … distinct from conceptual, intellectual learning (Ecker et al., 2012, p. 27). Because of the speed by which acupoint tapping can send activating and deactivating signals to brain regions involved with the treatment focus, tapping protocols are able to rapidly produce prediction errors. The craving that existed a moment ago no longer exists, even with the food still present or imagined. Such experiences lead to the permanent updating of existing knowledge structures and are a consistent feature in acupoint tapping treatments (Feinstein, 2021b).

### Other biological shifts explain benefits beyond targeted issues

Therapeutic goals such as reducing anxiety, depression, or anger—or changing early programming—are all served by interventions that generate signals which ease hyperarousal, stimulate executive brain regions, or revise existing cognitive structures. In addition to psychological issues, ACEs also lead to a range of serious physical health issues. Laboratory studies have identified several biological markers following tapping sessions that were not related to the purpose of the treatment yet which tend to have a positive impact on health. These non-specific benefits include favorable shifts in the production of stress hormones, gene expression, immunity, cardiovascular function, and brainwave ratios. Each will be briefly examined.

#### Stress hormones

In the first investigation of changes in cortisol levels following a tapping session, 83 study participants were randomly divided into three groups (Church et al., 2012). One received a 1-h EFT session; another a 1-h supportive counseling session; and the third, no treatment. Salivary cortisol levels were assessed for each participant before the session and, for those receiving treatment, half an hour after the treatment. While both treatment groups showed lowered cortisol levels, the decrease was nearly twice as large for the tapping group. Participants in the tapping group also reported greater decreases in psychological distress. This finding has been replicated in subsequent studies (Stapleton et al., 2020; Okyay and Ucar, 2023).

#### Gene expression

Among the most important medical discoveries in the second half of the twentieth century was that even if the gene for a particular trait is in the genome, whether or not that trait will appear often depends on a complex interaction of stress, age, diet, hormones, and environmental influences (Singh et al., 2018). Known as “gene expression,” a person’s external environment and internal chemistry can act as a gene’s on–off switch as well as the volume control when the switch is on. Favorable shifts in the *expression* of genes that influence health and brain activity is another area in which physiological changes have been shown to follow acupoint tapping sessions. This was first reported in a 2016 pilot study with four participants who received a 1-h EFT session. Changes were found in gene expression that is associated with desirable shifts in learning, emotional regulation, neuroplasticity, synaptic connectivity, and building white matter in the brain (Maharaj, 2016). Two years later, investigators of a 10-session EFT program treating 16 veterans with PTSD and related health conditions reported that the treatment favorably increased the expression of six genes, including one that influences immunity (Church et al., 2018b).

#### Immunity and cardiovascular function

In addition to the finding that changes in gene expression that affect immunity follow tapping treatments, direct pre/post-treatment measures of immunity have also been conducted. Bach et al. (2019) found significant increases in immune responsiveness based on salivary immunoglobulin A (SigA) in 31 individuals who participated in a 4-day EFT training workshop. The same study also found statistically significant improvements in blood pressure and resting heartrate as well as other trends suggesting improvement in cardiovascular function.

#### Optimizing brain wave ratios

During an EFT session with a 51-year-old woman who had residual symptoms from a traumatic brain injury, EEG patterns showed increasing relaxation and a marker for feeling “centered” as the treatment progressed (Craig et al., 2009). In a study of the use of EFT following trauma, EEG monitoring revealed reduced arousal in the right frontal cortex of nine victims of motor vehicle accidents following treatment (Swingle et al., 2005). EFT treatments in patients with seizures resulted in desirable increases in the amplitude of the sensory motor rhythm of the sensory motor cortex (Swingle, 2010). A study by Lambrou et al. (2003) demonstrated that a single EFT session for claustrophobia led to the normalization of theta waves and reductions in muscular tension in all four study participants.

Neurofeedback researchers Gary Groesbeck and Donna Bach, have conducted hundreds of EEG readings *during* acupoint tapping sessions, utilizing digital EEG equipment. A digital EEG allows for real-time monitoring of brain wave changes during tapping sessions and can even be projected on a screen for an audience to observe. During one audience demonstration observed by this author, Groesbeck and Bach were able to describe how the brainwave patterns revealed disturbances when the client initially focused on stressful thoughts and how these patterns shifted throughout the session, corresponding with reduced distress, improved left–right hemisphere synchronization, and an overall optimization of brain wave ratios.

#### Specific and generic health benefits

Non-specific therapeutic factors, such as a clinician’s empathy or a client’s positive expectations may influence the effectiveness of any therapeutic approach regardless of its theoretical orientation or specific techniques. Analogously, non-specific biological markers, as discussed above, may benefit a client’s health even when not a focus of the treatment. Biological markers identified following energy psychology treatments—involving stress hormones, gene expression, immunity, cardiovascular function, and brainwave ratios—may have health benefits that are not identified in outcome assessments that focus on the treatment goals. Some energy psychology treatments, however, have attempted to directly bring about improvement in specific health conditions and have measured health outcomes. Among those that demonstrated statistically significant health benefits include improvements in tension headaches (Bougea et al., 2013), pulmonary conditions (Babamahmoodi et al., 2015), fibromyalgia (Brattberg, 2008), traumatic brain injury (Church et al., 2016), side effects of chemotherapy (Baker and Hoffman, 2015), and controlling blood glucose levels in diabetic patients (Hajloo et al., 2014).

## Neurology-based strengths of acupoint tapping in treating ACE-related conditions

Difficulties encountered by individuals who have undergone severe or multiple ACEs include a spectrum of physical and psychological symptoms. Underlying these are brain mechanisms that may point toward shared neurological factors that can be targeted by treatment strategies. A formulation that is useful for understanding energy psychology treatments involves three neural networks which may become impaired during sensitive developmental periods.

### Neural networks affected by severe or multiple ACEs

Weems et al. (2021) explain that these brain networks, which are connected by function rather than location, are based on the “coordinated activation of multiple, regionally disparate areas of the brain” (p. 192). They include the *salience*, *central executive*, and *default mode* networks (p. 193). Some of the functions of each that are directly related to individuals who have experienced severe or multiple ACEs follow.

#### The salience network

The linked brain regions of the salience network play a major role in detecting and processing threat-relevant stimuli. An overactive *salience network* may lead to hyperarousal, emotional numbing, or both. This network involves the threat detection role of the amygdala, the threat interpretation role of the insula, the threat response roles of the amygdala and hypothalamus, the inhibitory role of the ventromedial prefrontal cortex (vmPFC), the fear extinguishing role of the ventral striatum, and the memory processing role of the anterior cingulate. Sheridan and McLaughlin (2020) emphasize the dynamic interplay among these brain regions, explaining for instance how the amygdala “underlies the acquisition and expression of conditioned fear” while the vmPFC “directly inhibits the amygdala,” decreasing the strength of learned fear and promoting fear extinction (p. 269).

The salience network is often involved in the treatment of maladaptive coping strategies associated with ACEs because of its roles in threat detection, interpretation, response, and processing. Prolonged exposure is among the most utilized techniques for addressing distortions in threat processing (McSweeney et al., 2022). With repeated exposure to the cue (and often progressing to more threatening cues), the new neural pathways no longer predict harm in the presence of the cue. The brain habituates to the cue’s appearance, with the result that the sensations, emotions, and troubling thoughts that had been associated with the cue are no longer evoked. As the therapy progresses, and the salience network becomes less reactive to the cue, clinically favorable changes in the central executive network may also occur, sometimes spontaneously, sometimes aided by cognitive interventions.

#### The central executive network

These linked regions are associated with attention allocation, rule-based problem-solving, manipulating information in working memory, decision making, and the self-regulation of emotions. Distress tolerance, the interpretation of trauma reminders, and the defensive use of cognitive distortions are also in the domain of this network. Components of the *central executive network* include various regions of the prefrontal cortex as well as the anterior cingulate cortex, which is uniquely situated between the limbic system and the prefrontal cortex, with direct connections to both. Fear structures are coded in the central executive network, as explained by (Watkins et al., 2018):

Fear is represented in memory as a cognitive structure that includes representations of the feared stimuli, the feared responses, and the meaning associated with the stimuli and responses to the stimuli… Fear structures may become problematic when the association between stimulus elements do not accurately reflect the real world, physiological and escape or avoidance responses are induced by innocuous stimuli, responses that are excessive and easily triggered interfere with adaptive behavior, and safe stimulus and response elements are incorrectly associated with threat or danger (p. 5).

#### The default mode network

These linked regions are active during wakeful rest and may involve intrusive thoughts, daydreams, and ruminating on past experiences. When the salience network or the central executive network is active, the *default mode network* is less engaged, but during periods of their minimal activation, the default network system produces spontaneous thoughts, retrieves autobiographical memories, activates daydreams, reexperiences past traumas, and interprets them. This network involves the attention focusing role of the posterior cingulate cortex, the role of the hippocampus in activating and processing memories, the role of the medial temporal lobe in mediating emotional processes such as sensitivity to threatening cues, and the threat modulation role of the ventromedial prefrontal cortex. Alterations in the function of the default network caused by ACEs include distorted retrieval of autobiographical memories, exaggerated distress from recollections of past trauma, and biased interpretations of interpersonal experiences.

The default mode network manages the way incoming information is processed (Brandman et al., 2021) and “facilitates construction of mental models” (Buckner, 2013, p. 351). The adaptive functions of managing incoming information and formulating mental models based on that information are obvious, but so is the possibility of cognitive distortions biasing both processes. The default mode network can also be hijacked by other parts of the brain. For instance, if neural regions involved in depression, such as the medial prefrontal cortex and the subgenual anterior cingulate, interact with the default mode network in this manner, “resting thought becomes entirely consumed with negative ruminations” (Forster, 2017).

#### Implications

An implication of the model presented by Weems et al. (2021) is that bringing about changes in these three neural networks will be of benefit to individuals who been negatively impacted by ACEs. Acupoint tapping protocols, as mind–body approaches, have advantages in addressing all three networks. While the content of the biographical material and its neurological consequences will vary depending on whether the ACEs were based on abuse or other harm, threat of harm, neglect, or a combination (Sheridan and McLaughlin, 2020), energy psychology works with that content in similar ways.

### How acupoint tapping can impact the *salience network*

The salience network can become chronically overactive following intense or ongoing childhood experiences involving perceived threat or actual harm. The consequent distortions in the detection, interpretation, and processing of threat may lead to hyperarousal, emotional numbing, or both. Whether in hyperarousal or numbing, estimations of threat even when no longer in the dangerous situation are exaggerated and responses to perceived threat are often maladaptive.

Exposure therapy is the most frequently recommended approach for treating these sequelae of traumatic experiences (McSweeney et al., 2022), and exposure is an essential component of acupoint tapping protocols. However, exposure techniques that simultaneously utilize tapping involve a different process neurologically than conventional exposure (Feinstein, 2022).

In conventional exposure, two independent processes occur in sequence. First, because the threat inducing cues are not followed by the anticipated tangible threat or harm, the fear structure accommodates the more recent experience that the cue is innocuous. Second, only after this has been accomplished, can the brain begin to habituate to the cue’s appearance, so the sensations, emotions, and troubling thoughts that had been associated with the cue no longer occur.

When acupoint tapping accompanies the exposure, the two processes occur simultaneously. Rather than reducing the threat reaction through repetition and habituation *after* the cue has been recategorized as harmless, the threat reaction is rapidly attenuated by the deactivating signals the tapping sends to the limbic system. This turns off the threat response while the memory or cue is still mentally active. The process of tapping while bringing to mind a disturbing memory soothes the nervous system at the same time the thoughts or images are provoking it. This facilitates both the elimination of threat based reactions to the cue as well as its recategorization as being safe.

The first somatic intervention to identify advantages over conventional exposure was Eye Movement Desensitization and Reprocessing (EMDR). Rogers and Silver (2002) noted that “previous research suggests that repeated brief exposures only result in fear decrement when stimulus intensity and arousal are both low. Yet EMDR uses very brief (20-30-s) exposures [even though] stimulus intensities are high, since clients are asked to start by focusing on the most distressing scene” (p. 49). Building on Rogers and Silver’s observations, Feinstein (2022) identified differences between conventional exposure and exposure that utilizes tapping as including:

1. Brief exposure (10–15 s in each round) is considered in conventional exposure treatments to be effective for low levels of arousal but not for highly distressing stimuli while brief exposure along with acupoint tapping has been found to be effective with high as well as low levels of arousal.
2. Prolonged exposure is generally required for conventional treatments of highly distressing stimuli while prolonged exposure is not required with acupoint tapping protocols to obtain desired clinical outcomes even with highly distressing stimuli.
3. Guidelines for conventional exposure require that clients keep their attention on traumatic cues without shifting attention to other thoughts while attention during effective tapping sessions can shift among traumatic memories or cues and other thoughts, beliefs, physical sensations, emotions, or expectations.
4. Conventional exposure is recommended for fear and anxiety but is not considered effective in the treatment of guilt or other complex emotions that require higher order cognitive constructs while emotions such as guilt, shame, and grief have responded to acupoint tapping combined with psychological exposure.

The most important postulated difference between conventional exposure and exposure with acupoint tapping, however, involves the way original fear memories are processed. Edna Foa and Richard McNally, two of the pioneers of exposure treatments, explained that based on “abundant evidence…fear reduction does not involve the weakening of associations *per se*, but rather involves the formation of new associations [that] override the influence of pathological ones” (Foa and McNally, 1996, p. 339). Since that time, pharmaceuticals first, and then highly specific cognitive procedures, demonstrated a second way the brain updates itself, in addition to overriding old learnings. This involves fully “depotentiating” the neural networks that retain the old learning at the synaptic level. Monfils et al. (2009) explain that comparisons of conventional extinction methods with those that eliminate rather than override old associations “engage different mechanisms in the lateral amygdala and lead to a drastically different behavior outcome” (p. 953). By instantly sending deactivating signals to the limbic system that has been put into a state of high arousal by the psychological exposure, acupoint tapping is believed to utilize this second set of mechanisms, leading to the unusually rapid and durable change demonstrated in the clinical trials (Feinstein, 2022).

### How acupoint tapping can impact the *central executive network*

As the salience network becomes less reactive, a cascading series of shifts spontaneously occur in the central executive network which incorporate the transformed relationship with the previously threat inducing cues. While this may occur with no volition, acupoint tapping protocols can also directly address activity in the central executive network. Sise and Bender (2022) distinguish between everyday beliefs that may interfere with effective coping and deeper “core beliefs” that maintain lifelong patterns of thought and behavior. They outline acupoint tapping procedures which fall in the domain of the central executive network for working with both types of belief. For instance, after identifying a belief that is exerting a negative impact on the person’s life and noting any signs of bodily distress when bringing that belief to mind, the procedures involve tapping on (a) the identified sensations, (b) resistance to changing the belief, (c) statements of intention to change it, and (d) statements that articulate the new more adaptive belief.

What acupoint tapping adds to this sequence of steps, which might be typical of any CBT-oriented approach is, first, that the tapping decreases the intensity of sensations that can interfere with changing the belief. But tapping also seems to embed the intention and statements more deeply into the nervous system (Feinstein and Eden, in press). Acupoint tapping can be adapted into any modality that is designed to overcome cognitive distortions, revise maladaptive behavioral strategies, and update mental schemas based on more recent experiences.

### How acupoint tapping can impact the *default mode network*

Acupoint tapping protocols can also be applied for bringing about changes in the default mode network which tends to come online when the salience and central executive networks are less active. The ruminations of the default mode network are ways the brain reviews experience, incorporates new information, and revises mental models. Distortions in ruminations and information processing associated with ACEs tend to maintain symptoms and maladaptive behaviors. The therapeutic procedures for working with the default mode network, such as relaxation, mindfulness, and guided imagery techniques themselves operate in the realm of the default mode network.

Acupoint tapping can be combined with each of these techniques. Practitioner surveys suggest that tapping combined with another mental health intervention increases the impact of the accompanying approach (Feinstein, 2021a). Gallo (2015), for instance, reports the advantages of integrating tapping with mindfulness procedures. Meanwhile, tapping alone has been shown to be more effective than mindfulness alone. For instance, a study of university students comparing EFT and mindful breathing found that the EFT group reported greater increases in enjoyment, hope, and pride and greater decreases in anger, anxiety, and shame than did the mindful breathing group (Fox, 2013). The actions of interventions that impact the other neural networks may also cascade into the default mode network. For instance, cognitive interventions that address distortions in memory and information processing in the central executive network will change the content that emerges in the default mode.

## Recommendations for integrating acupoint tapping into the treatment of individuals with severe or multiple ACEs

The World Health Organization’s International Classification of Diseases (11th Edition) provides this definition of “Complex PTSD”:

Complex post traumatic stress disorder (Complex PTSD) is a disorder that may develop following exposure to an event or series of events of an extremely threatening or horrific nature, most commonly prolonged or repetitive events from which escape is difficult or impossible (e.g., torture, slavery, genocide campaigns, prolonged domestic violence, repeated childhood sexual or physical abuse). All diagnostic requirements for PTSD are met. In addition, Complex PTSD is characterized by severe and persistent (1) problems in affect regulation; (2) beliefs about oneself as diminished, defeated or worthless, accompanied by feelings of shame, guilt, or failure related to the traumatic event; and (3) difficulties in sustaining relationships and in feeling close to others. These symptoms cause significant impairment in personal, family, social, educational, occupational or other important areas of functioning.^1^

Treatment guidelines for complex PTSD (cPTSD) are much more developed than treatment guidelines for individuals whose history includes severe or multiple ACEs and provide a starting point for developing guidelines in using energy psychology with this population. In fact, most of the clinical trials providing information used in developing early guidelines for treating cPTSD specified a history of childhood physical and/or sexual abuse as an inclusion criterion (De Jongh et al., 2016).

### ISTSS guidelines for treating cPTSD

The International Society for Traumatic Stress Studies has been developing the most widely referenced practitioner guidelines for treating cPTSD, drawing upon the entire range of evidence-based psychotherapies (International Society for Traumatic Stress Studies Guidelines Committee, n.d.). An anthology, *Effective Treatments for PTSD: Practice Guidelines from the International Society for Traumatic Stress Studies* (Forbes et al., 2020), provides practitioners with detailed guidance for applying the ISTSS formulations to the treatment of PTSD. The book’s chapter on cPTSD (Cloitre et al., 2020) frames the treatment of cPTSD according to a basic set of assumptions and clinical orientations that are conveyed to the client:

A fundamental principle guiding both the assessment and treatment of individuals with cPTSD is the assumption that many of the symptoms resulting from sustained and multiple forms of interpersonal trauma begin as adaptive efforts under conditions of extreme adversity. Dissociation and emotional numbing are protective reactions to overwhelming experiences. Avoidance of relationships and intimacy can be strategies for staying physically and emotionally safe. Mistrust and aggressive behaviors such as those that occur in combat or environments of chronic violence (e.g., multi-generational civil war) are necessary for survival. These reactions transition from adaptive to nonadaptive when the traumatic threats no longer are present but the affective, cognitive, and behavioral adaptations continue creating a mismatch between the environment and responses to it.

During the assessment and throughout all phases of the treatment process, the therapist instills hope by describing how the treatment will address the specific problems that the patient is presenting, by identifying the specific personal and relational resources the patient is bringing to the treatment, and by describing the collaborative nature of decision making in the therapy. The therapist listens carefully and without assumptions about which problems bother the patient most and respects the individual’s interests and preferences. Emphasis on the collaborative nature of the work, responsiveness to the patient’s concerns, and flexibility in implementing the treatment all support the therapeutic alliance, which has been consistently demonstrated as a significant contributor to engaging, retaining, and achieving positive outcomes with adults with a wide range of psychosocial problems (pp. 366–367).

Establishing and maintaining a strong therapeutic alliance is an ongoing clinical objective that must be approached with a sensitivity to the fact that people with a history of complex trauma may have experienced “sustained victimization, deception, exploitation, or betrayal by trusted others” (p. 366). Even the informed-consent process should be meticulous about issues such as patient rights, the responsibilities of the therapist, the procedures that will be used, and limitations of the treatment, potential outcomes, confidentiality, and HIPAA guidelines. Within this framework, Cloitre et al. outline three tasks that must be accomplished for the successful treatment of cPTSD. They include assessment, treatment planning and implementation, and follow-up care.

#### Assessment

In addition to exploring symptoms and history in order to establish an informed diagnosis, the current life circumstances of a person with suspected PTSD or ACEs should be reviewed for safety and stability, and a safety plan established if needed. When the back home environment is unsafe or unstable, social service support or related interventions may be required if psychotherapy is to be effective. Another challenge during this phase is to distinguish between cPSTD and borderline personality disorder (BPD). While these conditions both involve difficulties in affect regulation, self-concept, and interpersonal relationships, the way these problems manifest are different for each, and the two are distinctly different disorders with differing diagnostic criteria (Hyland et al., 2019).

#### Treatment planning and implementation

Of the six cPTSD symptom clusters identified in the literature, three are specific to traumatic injury (reexperiencing, avoidance, and heightened sense of threat) and three are related to disturbances in self-organization (emotion dysregulation, negative self-concept, and interpersonal difficulties). The largest part of the cPTSD chapter by Cloitre et al. (2020) focuses on considerations involving the planning and implementation of treatment for these symptom clusters. While it is beyond the scope of this review to adequately recap their thoughtful, evidence-informed discussion, several takeaways can be distilled and briefly mentioned:

##### Multimodal approach

The interventions that are most effective with one of the cPTSD symptom clusters may not be as effective with others, and client differences may also lend advantages to one approach for a particular symptom over others. For instance, therapies that focused primarily on trauma were less effective for individuals with histories of childhood trauma than for those whose traumas were experienced only while adults. A multimodal approach takes advantage of the availability of a variety of validated trauma memory processing therapies and provides “flexibility with regard to the selection and implementation of interventions so that they can be organized to be responsive to the presenting problems, needs, and preferences of patients” (Cloitre et al., 2020, p. 370).

##### The most important cPTSD symptoms to target

Given the complexity of the conditions that grow out of severe traumatic experiences or ACEs, the question of where to focus is paramount in formulating and implementing a treatment approach. While the treatment goals are framed in collaboration with the client, research findings can inform the process. Four analyses using large nationally representative samples found that negative self-concept was the most central feature, followed by emotional dysregulation (Knefel et al., 2019). Related to difficulties with emotional regulation were interpersonal problems. These three symptom clusters—negative self-concept, emotional dysregulation, and interpersonal difficulties—were more central for treating cPTSD than avoidance, reexperiencing, and heightened sense of threat.

##### The order of interventions

After establishing treatment goals collaboratively set by the client and the therapist, a logical sequence of objectives might for instance, include “first, interventions to ensure safety and enhance personal, social, and environmental resources and self-regulation capacities; second, interventions for trauma memory processing (TMP); and third, interventions for generalizing therapeutic gains to daily life” (Cloitre et al., 2020, p. 374). Addressing these objectives in a sequence not only allows more advanced understanding and skills to build on more primary ones, it also focuses the client’s time and energy on manageable doses of new learning and behavioral practices. On the other hand, rigidly adhering to a predetermined sequence may not accommodate patient differences and preferences, such as if the client wishes to immediately focus on an intrusive memory or on nightmares that are interfering with sleep and daily functioning. It may also be necessary with some clients, particularly in residential treatment settings, to combine multiple intervention components simultaneously, such as in the treatment of cPTSD and major depressive disorders, substance abuse disorders, or other co-morbid conditions.

##### Length and tempo of treatment

Given the complexities of cPTSD and ACEs, treatment length will be highly variable. While many clients will achieve substantial symptom relief and other gains within a few months, others may need more than a year for fundamental and durable changes, often followed by periodic check-ins. Within the therapy sessions, tempo is another issue. Cloitre et al. (2020) elaborate:

It is important to choose a tempo that matches the client’s capacity for learning and to engage in a deliberate pace that neither pushes for overly rapid results nor slows to a standstill due to fear, dysphoria, or other emotional or interpersonal problems. Helping the client to make steady progress even when crises or “stuck points” occur provides a corrective learning experience that represents an important contrast with previous life experiences (p. 378).

Complicating the treatment’s ability to maintain a productive tempo is the fact that individuals with cPTSD or ACEs are more likely to experience new crises or ongoing exposure to major life stressors such as domestic violence or family members who are in crisis. Cloitre et al. (2020) advise maintaining a “coherent treatment focus based on the treatment plan, while also flexibly moving between the two primary tasks of cPTSD treatment: processing memories of past traumas and dealing with current stressors or life activities” (p. 378).

##### Concurrent problems and collaboration with other providers

Clients with cPTSD or severe or multiple ACEs often present with chronic health disorders or physical injuries arising, for instance, from sexual assault or other forms of violence. Collaboration with the client’s other health care specialists can lead to an integrated treatment plan that can be tracked over time. Consistent communication among providers and with the client counters the risk of the client experiencing what might appear to be conflicting treatment approaches. Cloitre et al. (2020) also suggest that there may be a need for mental health professionals to “provide respectful and sensitive education to other providers involved in the client’s care…concerning the clients’ possible cues and triggers, attitudes, and concerns (e.g., distrust of authority figures), and symptoms (e.g., dissociation, reduced attentional capacities) that will facilitate collaborative and effective management of the client’s multiple health problems” (p. 379).

##### Follow-up care and reintegration

The final phase of therapy involves planning that maintains gains, anticipates the possibility of setbacks, and modifies former life structures and adaptations. Cloitre et al. (2020) describe the challenges:

Due to the chronicity and severity of their traumas as well as their symptoms, individuals with cPTSD may have become disconnected from or never participated in family or community life, have limited social support and few relationships, and have limited education or employment prospects. Common underlying factors driving these circumstances are a sense of being somehow “damaged” or “different” from members of the surrounding community [due to] limitations or a deterioration in their functional capacities related to relationships, social engagement, and work (pp. 367–368).

Follow-up care may involve continued contact with the therapist via tune-up sessions and, if necessary, a return to treatment during times of stress. Additional recommendations might include peer empowerment groups and resources that support education, employment, housing, health care, parenting, or addiction recovery. Of course any model for treating cPTSD and developmental trauma is rarely simple or straightforward, so guidelines should be flexibly applied.

#### EFT guidelines for treating cPTSD

EFT Guidelines for treating PTSD were derived from literature reviews and a detailed survey that was responded to by 448 EFT practitioners (Church et al., 2018a). The drafted guidelines were then assessed and refined by representatives of the major institutions providing training or supporting research for the approach. The Guidelines recommend:

…a stepped-care model, with five treatment sessions for subclinical PTSD, 10 sessions for PTSD, and escalation to intensive psychotherapy or psychopharmacology or both for nonresponsive patients and those with developmental trauma. Group therapy, social support, apps, and online and telemedicine methods also contribute to a successful treatment plan (para. 1).

“Escalation to intensive psychotherapy” would also apply to cPTSD and severe or multiple ACEs. The EFT Guidelines do not specifically reference ACEs but rather address the pertinent dynamics by discussing developmental trauma:

In developmental trauma, traumatic events occur while the child’s brain, personality, attachment patterns, schemas, and internal models of relationships were still forming. As a result, the neural circuitry of PTSD was firing and wiring in the emotional midbrain during a crucial developmental stage, embedding patterns of stress into the child’s interpersonal neurobiology and attachment templates (para. 41).

The Guidelines imply that EFT can be applied to overcoming ACEs because it “has been effective with complex personality and relational difficulties that are rooted in childhood trauma” (para. 41). The Guidelines note that even though developmental trauma is often coded non-verbally, it may even have occurred prior to the acquisition of language. EFT protocols address nonverbal material directly by tapping on acupoints while sensations and emotions related to traumatic symptoms are mentally active, even if the formative memories cannot be accessed. In practice, reducing the intensity of the sensations and emotions often allows repressed memories to emerge. The Guidelines recommend that in this escalated level of treatment, timeframes should be adjusted as the therapy progresses.

## Recommendations for using energy psychology with adults whose symptoms trace to ACEs

While energy psychology has its own professional organization (the Association for Comprehensive Energy Psychology, https://www.energypsych.org/), journal,^2^ and a substantial scientific literature (an up-to-date database is maintained at https://www.energypsych.org/researchdb8c71b7), its distinguishing technique, acupoint tapping, is flexible and can be integrated into any therapeutic modality or orientation. Acupoint tapping itself is psychologically atheoretical. The procedure sends electrical signals that may activate or deactivate specific areas of the limbic system, prefrontal cortex, or other brain regions. Where these signals are sent depends on the words, images, thoughts, and feelings brought to mind while doing the tapping, which grow out of the therapist’s theoretical orientation and serve the treatment goals as jointly conceptualized by the therapist and client.

Severe or multiple ACEs and the experiences that lead to cPTSD bring about many overlapping trauma-induced symptoms. For this reason, the existing ISTSS and EFT guidelines for treating cPTSD provide a preliminary foundation for utilizing acupoint tapping to treat the issues that result from ACEs, at least until more ACE-specific guidelines are developed. The assumptions delineated by Cloitre et al. (2020), above, such as that symptoms often trace to adaptive efforts under conditions of extreme adversity, that instilling hope is an underlying orientation regardless of specific techniques, and that therapeutic goals and decisions are done on a collaborative basis all hold when ACEs are involved. In addition, the challenges in forming an effective therapeutic alliance with individuals who have experienced sustained deception, exploitation, or betrayal may also apply when severe or multiple ACEs are part of the client’s history.

What acupoint tapping adds to treatments involving ACEs is not so much to provide altogether new methods but to make existing methods more effective. The earlier discussion contrasting conventional exposure techniques with tapping *during* exposure serve as a prototype for the integration of acupoint tapping with other treatment modalities. Acupoint tapping can be utilized in these ways to accomplish the three clinical tasks identified by Cloitre et al. (2020).

### Assessment

In addition to the assessment considerations outlined by Cloitre et al. (2020) are special challenges for an energy psychology approach. In the early phases of treatment, the client is assessing the therapist just as the therapist is assessing the client. One of the requirements for an approach that may be as unfamiliar, no less odd-seeming, as acupoint tapping, is to explain where it fits into the treatment. As early as prudent, while staying sensitive to the client’s possible mistrust and skepticism, the effectiveness of acupoint tapping should be demonstrated. Identifying a minor fear or anxiety, such as around an upcoming conversation or task, is a good target for the first tapping experience. Once tapping has been introduced, it can be applied so any moments of distress during history taking can be rapidly soothed with the tapping, further establishing it as a viable treatment modality.

### Treatment

Therapeutic outcomes have been shown to markedly improve by keeping the client engaged in the ever-evolving treatment plan (Norcross and Cooper, 2021). Consistent with other guidelines, built into energy psychology protocols are frequent assessments of progress. This allows a personalizing of the therapy. By taking progress readings after every round of tapping, the treatment is continually being adjusted according to the client’s needs, preferences, and symptom responsiveness. As the energy psychology treatment progresses, early traumatic experiences can be revisited in greater detail, using acupoint tapping to reduce distress in a manner that facilitates the limbic system’s processing of unresolved emotional material. By incorporating tapping techniques along with cognitive reframing and guided imagery, clients can strengthen and reinforce new cognitive and behavioral patterns that align with desired outcomes. In situations where the therapeutic process evokes overwhelming anxiety, sadness, fear, or anger, acupoint tapping can immediately be applied to downregulate the autonomic nervous system, reduce distress, and restore the ability to focus and stay present. This is one of the more useful benefits of the approach in working with traumatized individuals. Cloitre et al. (2020) also discuss the importance of providing corrective learning moments that represent significant contrasts with previous life experiences. Acupoint tapping that immediately reduces the response to threat inducing cues creates such contrasting experiences.

### Follow-up care and reintegration

Throughout the therapy, clients are encouraged to reinforce the gains from the sessions by tapping between sessions and also to apply tapping as new emotional challenges arise. Continuation of these practices is generally part of the post-therapy plan, and various external resources are available. For instance, a mobile app that guides users in applying acupoint tapping protocols for anxiety and stress was investigated in a large-scale study including 270,461 app users and found highly significant (*p* < 0.001) symptom reduction (Church et al., 2020). Toward the end of therapy, imagining emotional challenges that can be anticipated and tapping on them prepares the client for trials likely to be encountered post-therapy. Combining tapping with this guided imagery seems to enhance the power of the imagery in shaping self-concept and future behavior.

#### Recap

Acupoint tapping can be integrated with other evidence-based practices at each stage in the treatment of clients who have undergone severe or multiple ACEs.

## Discussion

Cloitre et al. (2020) caution that “cPTSD is a new diagnosis and there is to date no direct evidence about how to treat it” (p. 369). The ISTSS cPTSD Guidelines suggest that when empirical evidence is limited, the highest likelihood of achieving desired outcomes grows out of a meeting of the therapist’s expertise with the client’s values, preferences, and previous successes and failures in dealing with persistent symptoms. Meanwhile, sound guidelines have been evolving and informing practice. This discussion of guidelines for applying energy psychology in the treatment of clients who have experienced severe or multiple ACEs is also preliminary, yet it provides a framework for clinicians working with this potentially challenging population.

An implication of this paper is that therapists who work with clients whose symptoms are related to ACEs can increase their effectiveness by incorporating acupoint tapping into their existing clinical repertoire. Conveniently, acupoint tapping is relatively easy for clinicians to learn and incorporate. A total of 48 “core techniques” are taught in one of the most well-regarded tapping certification programs.^3^ Among them, beyond the actual tapping procedures, are techniques for identifying core issues, uncovering specific events linked to the origins of psychological difficulties, and reducing emotional intensity. Experienced clinicians will find that many of these necessary skills are already in their repertoire.

A welcome and often unexpected outcome of introducing acupoint tapping protocols into psychotherapy is that it seems to produce a favorable shift in the therapist-client relationship. For instance, a phenomenological study based on interviews with 16 seasoned therapists of varying clinical orientations who had introduced EFT into their already established practices found that all 16 independently reported that the new technique had the effect of enhancing the therapeutic alliance with their clients (Nairn, 2020). The therapists speculated that tapping facilitated rapid and tangible shifts, providing direct subjective evidence of therapeutic progress. Additionally, they found that tapping, when performed along with the client (which is standard practice), demonstrated their active involvement, introduced a ritualistic element that fostered resonance between therapist and client, and enhanced their intuitive understanding of the client’s concerns.

## Conclusion

Individuals who have undergone severe or multiple ACEs may experience a variety of challenging psychological, health, and adjustment difficulties. While empirically validated therapies for the spectrum of conditions related to ACEs are not yet available, the neurological and psychological underpinnings relevant for treatment are being identified and studied, and preliminary treatment guidelines are suggested. Energy psychology protocols, with their use of acupoint tapping, show promise for enhancing the effectiveness of more conventional clinical techniques.

## Author contributions

The author confirms being the sole contributor of this work and has approved it for publication.

## Footnotes

^1^https://icd.who.int/en

^2^https://energypsychologyjournal.org/

^3^https://EFTUniverse.com
